# Auxiliary Diagnosis of Dental Calculus Based on Deep Learning and Image Enhancement by Bitewing Radiographs

**DOI:** 10.3390/bioengineering11070675

**Published:** 2024-07-02

**Authors:** Tai-Jung Lin, Yen-Ting Lin, Yuan-Jin Lin, Ai-Yun Tseng, Chien-Yu Lin, Li-Ting Lo, Tsung-Yi Chen, Shih-Lun Chen, Chiung-An Chen, Kuo-Chen Li, Patricia Angela R. Abu

**Affiliations:** 1 Department of Periodontics, Division of Dentistry, Taoyuan Chang Gung Memorial Hospital, Taoyuan City 32023, Taiwan; an840802@cgmh.org.tw (T.-J.L.); 700713@cgmh.org.tw (Y.-T.L.); 2 Department of Program on Semiconductor Manufacturing Technology, Academy of Innovative Semiconductor and Sustainable Manufacturing, National Cheng Kung University, Tainan City 701401, Taiwan; m28121562@gs.ncku.edu.tw; 3 Department of Electronic Engineering, Chung Yuan Christian University, Taoyuan City 32023, Taiwan; s11020135@cycu.edu.tw (A.-Y.T.); s11020102@cycu.edu.tw (C.-Y.L.); s11020101@cycu.edu.tw (L.-T.L.); 4 Department of Electronic Engineering, Feng Chia University, Taichung City 40724, Taiwan; tsungychen@fcu.edu.tw; 5 Department of Electrical Engineering, Ming Chi University of Technology, New Taipei City 243303, Taiwan; 6 Department of Information Management, Chung Yuan Christian University, Taoyuan City 320317, Taiwan; kuochen@cycu.edu.tw; 7 Ateneo Laboratory for Intelligent Visual Environments, Department of Information Systems and Computer Science, Ateneo de Manila University, Quezon City 1108, Philippines; pabu@ateneo.edu

**Keywords:** dental calculus, image enhancement, YOLOv8, bitewing radiograph, medical image

## Abstract

In the field of dentistry, the presence of dental calculus is a commonly encountered issue. If not addressed promptly, it has the potential to lead to gum inflammation and eventual tooth loss. Bitewing (BW) images play a crucial role by providing a comprehensive visual representation of the tooth structure, allowing dentists to examine hard-to-reach areas with precision during clinical assessments. This visual aid significantly aids in the early detection of calculus, facilitating timely interventions and improving overall outcomes for patients. This study introduces a system designed for the detection of dental calculus in BW images, leveraging the power of YOLOv8 to identify individual teeth accurately. This system boasts an impressive precision rate of 97.48%, a recall (sensitivity) of 96.81%, and a specificity rate of 98.25%. Furthermore, this study introduces a novel approach to enhancing interdental edges through an advanced image-enhancement algorithm. This algorithm combines the use of a median filter and bilateral filter to refine the accuracy of convolutional neural networks in classifying dental calculus. Before image enhancement, the accuracy achieved using GoogLeNet stands at 75.00%, which significantly improves to 96.11% post-enhancement. These results hold the potential for streamlining dental consultations, enhancing the overall efficiency of dental services.

## 1. Introduction

According to the oral health issue proposed by the World Health Organization (WHO) in 2023 [1], the proportion of the global population suffering from oral diseases is increasing annually, with nearly half of the population concentrated in low- and middle-income countries. This issue demonstrates the dilemma faced by many individuals unable to access preventive and therapeutic services for oral diseases. Furthermore, promoting oral health is crucial for healthy aging [2], as good oral health enhances the quality of life for older adults and reduces societal resource demands. In this context, the elevation of dental healthcare awareness becomes particularly important. In the field of dental medicine, medical digital imaging technology plays a crucial role [3], as it facilitates the introduction of more convenient and efficient treatment approaches. The utilization of medical imaging necessitates dentists to undergo requisite training to ensure their proficiency in mastering these novel techniques, a process that demands additional time and resources. Furthermore, the subjective interpretation of symptoms via digital imaging may lack standardized protocols, potentially resulting in disparate diagnostic outcomes for different dental practitioners. To mitigate these existing challenges, artificial intelligence deployment in image recognition has emerged as a viable solution [4]. This technology delivers precise and swift automatic identification results and aids dentists in conserving the valuable diagnosis time, enabling patients to access comprehensive treatment.

**Figure 1:**
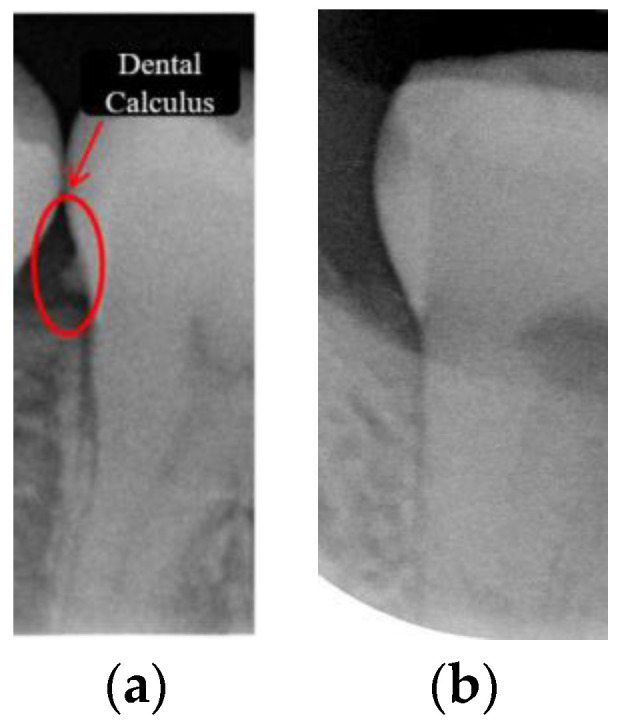
Dental calculus symptoms on a BW image: (**a**) dental calculus symptoms; (**b**) absence of dental calculus symptoms.

Dental calculus is recognized as a mineralized biofilm that comprises diverse calcium phosphate crystals, which may accumulate on the root surface both supra- and subgingivally [5]. A positive correlation between calculus deposits and periodontitis has been confirmed in multiple studies [6,7]. Despite clear evidence that the surface roughness of calculus alone does not initiate gingivitis, it is important to emphasize that calculus is consistently covered by an unmineralized layer of a viable biofilm [8], which is a primary factor in gingival inflammation [9]. The characteristics of dental calculus are not obvious in a BW image, as shown in Figure 1. The mechanical removal of subgingival plaque and calculus stands as the gold standard in the treatment of periodontitis [10]. While supragingival calculus can easily be seen and removed through sonic/ultrasonic instruments, subgingival calculus is invisible during intra-oral examination. Therefore, the identification of subgingival calculus is critical for the diagnostic process. Radiographic images have been widely used across various applications in the dental field [11]. Subgingival calculus is generally detected during periodontal probing, while radiographic examination can only show calculus on the proximal surfaces [12,13]. Meanwhile, Buchanan et al. [14] reported that the detection of dental calculus on the root surface by radiography had shown low sensitivity but high specificity, and a manual diagnosis can only detect dental calculus in 44% of the 275 datasets that truly have the condition. The detection ability can be increased to 82.2% by staining teeth with 1% methylene blue [15]. Galal et al. [16] observed that periapical films and intra-oral surveys showed comparable efficacy in calculus detection, with a particularly significant enhancement in detection when supplemented with radiographs. In comparison, other dental conditions like caries and periodontal disease have higher detection rates with manual methods. The detection rate for caries is approximately 70–85%, and for periodontal disease, it is around 65–80% [1]. Subgingival calculus documentation is crucial for periodontal assessment. Although clinical assessments are more common for detecting calculus, utilizing BW radiographs offers several advantages in calculus assessment. Firstly, BW images provide a comprehensive view of the tooth structure, allowing for a more thorough examination of interdental spaces where calculus often accumulates. Additionally, BW radiographs enable dentists to visualize areas that may be difficult to access during a clinical examination such as posterior regions. This enhanced visualization can aid in the early detection of calculus, leading to timely interventions and improved patient outcomes.

Considering the substantial workload faced by dentists who deal with a large volume of dental X-rays daily, this study integrates deep learning models and image-processing techniques to detect a single tooth in BW images and uses convolutional neural networks to classify dental calculus features. In the field of dentistry, several studies have focused on the AI analysis of dental X-ray images. For instance, the AI recognition of tooth positioning and caries was achieved using BW images [17], demonstrating an impressive accuracy of up to 90%. Convolutional neural networks were employed for detecting periodontal diseases [18,19] and periapical lesions [20] in periapical radiographs, yielding detection accuracies of 90%, 94%, and 96%, respectively. Various diseases were identified through image-segmentation and -enhancement techniques in DPR images in studies such as [21,22,23]. Object-detection models were utilized to detect features in DPR images [24], achieving an accuracy exceeding 90% in individually locating each tooth image. Convolutional neural networks (CNNs) were employed for symptom diagnosis [25]. Despite these studies, there is relatively limited research specifically addressing dental calculus symptoms in BW images. As mentioned above, numerous dental-related studies have now addressed this research gap by introducing the You Only Look Once (YOLO) object-detection model, providing more comprehensive and accurate information for diagnosis and treatment in the dental field. These studies typically utilize CNN classification based on transfer learning and image-processing techniques for symptom diagnosis in X-ray images, along with YOLOv8 and image-segmentation algorithms [26] for single-tooth segmentation in BW images. 

The database used in this study is the BW image database provided by the Chang Gung Memorial Hospital in Taoyuan, Taiwan. Each BW image was authorized by the patient for research purposes in this study and was approved by the IRB: 02002030B0. The presence of dental calculus in BW images was determined by dentists with over five years of experience. The dentists categorized the database into two groups: one containing 435 BW images with dental calculus and another containing 165 BW images without dental calculus. This database was utilized by the researchers for the image processing and training of deep learning models. The primary contributions of this study are as follows:

1. Using the YOLOv8 model as a method for BW image detection achieves an accuracy of 97%, representing a 2–14% improvement compared to the latest segmentation algorithms in current research.
2. Integrating a median filter and bilateral filter to reduce image noise effectively enhances the RoI while improving training accuracy. The accuracy can be enhanced to a 13–20% accuracy.
3. This study uses transfer learning and image enhancement to detect dental calculus symptoms and achieve 96.11% in GoogLeNet, which is 13.9% higher than the latest research.

## 2. Materials and Methods

**Figure 2:**
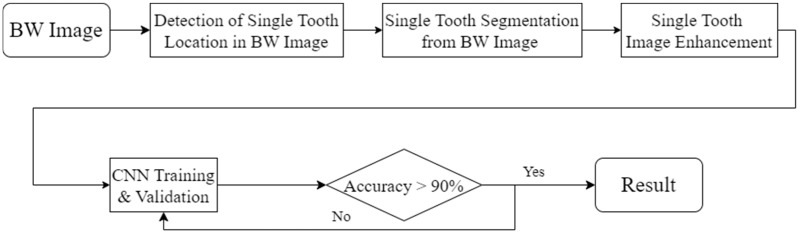
The flowchart used in this study.

This study uses image enhancement and deep learning to identify dental calculus symptoms in BW images. An accurate diagnosis requires the integration of these technologies. To achieve this goal, the research process was divided into four stages, as shown in Figure 2. The first is YOLO object detection. This stage can detect and record the position of the teeth in the BW image. In the BW image-segmentation stage, the tooth positions recorded in the previous stage are used to separate the teeth from the BW image. Next is the single-tooth image-enhancement stage, where techniques are employed to enhance the edges of dental calculus. Finally, training and evaluation are conducted using a classic convolution neural network. The ultimate objective of this study’s technology is to provide dentists with an auxiliary diagnostic model to reduce misdiagnosis risks and enhance hygiene education for patients.

### 2.1. Detection of Single-Tooth Location in BW Images

**Figure 3:**
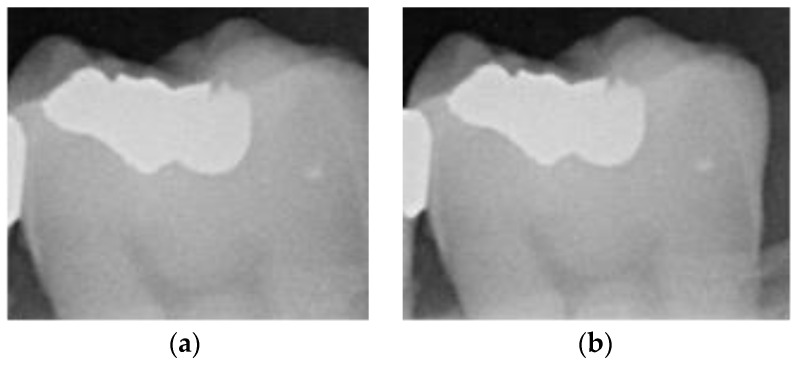
Image-annotation step preserves edges on both sides of the tooth: (**a**) less of the tooth edge is retained; (**b**) more of the tooth edge is retained.

Object-detection technology has become increasingly prominent in recent decades, due to advancements in machine learning and artificial intelligence playing a significant role in image processing [27]. Some well-known object-detection models, such as U-Net, Faster R-CNN, and the YOLO series, have been widely applied in various domains. However, the techniques used for dental calculus detection are still predominantly based on manual identification. Research on object detection in dental calculus remains relatively scarce. Therefore, this study chose to employ the YOLO model due to its promising performance and training potential. By leveraging YOLO, we aim to explore and improve the outcomes of object-detection training in the context of dental calculus. Therefore, the database was not easy to obtain, and this study used 435 BW images containing dental calculus, and each BW image contains at least one to three dental calculi. This study used a BW image database provided and annotated by dentists for the location of the dental calculus. During the research process, this study randomly selected X-rays from this database to create the training dataset. Before training the YOLO model, this study utilized the commonly used software tool “LabelImg” (version 1.8.1) for object annotation in YOLO. Additionally, dental experts assisted in annotating dental features for subsequent training purposes. In the image-annotation step of this study, rectangle boxes were used to label individual teeth in the BW images. This labeling method preserves larger surrounding features of the teeth. This is because placing the annotation box too close to the edges of the teeth may prevent YOLO training from fully extracting the features of dental calculus. Dental calculus typically appears on both sides of the teeth. The labeling method for teeth is illustrated in Figure 3. This study randomly selected 200 BW images that contain dental calculus and annotated 65% of the dataset images as the YOLO image training dataset, and the remaining 35% of the images were used as the validation dataset. The image dataset distribution is shown in Table 1. This study utilized YOLOv4, v5, v7, and v8 for image detection. In the YOLO models, there are slight differences in the architecture of each version. YOLOv4 uses CSPDarknet53 as the backbone, combined with PANet as the neck structure, and adopts the traditional YOLO head. YOLOv5 uses a customized CSPNet as the backbone, still employs PANet as the neck structure, and retains the YOLO head. YOLOv7 further improves the backbone by using an extended CSPNet and introduces E-ELAN as the neck structure, while the head part adopts a decoupled design. YOLOv8 [28] uses a mixture of experts’ models as the backbone, with an improved PANet as the neck structure, and the head part also uses a decoupled design to enhance detection performance, shown in Table 2. Before training, appropriate hyperparameters need to be set for each model, and these are shown, in detail, in Table 3. In this stage, the training results of YOLO will record the coordinates of detected teeth. These tooth coordinates will be utilized in subsequent stages for the segmentation of individual teeth in BW images through algorithms developed in this study. After multiple experiments and evaluations, this study found that different YOLO models require varying epoch lengths. Therefore, this study set up interrupts during YOLO training to automatically adjust algorithm parameters based on the results to achieve optimization.

### 2.2. Single-Tooth Segmentation from BW Images

BW images provide valuable information for dentists to evaluate the alignment, structure, and spacing of teeth. Unlike symptoms such as implants or root canals, dental calculus typically forms on the proximal surfaces of teeth, where its characteristics are not readily apparent and require additional image-processing methods for enhancement. However, directly enhancing dental calculus symptoms on BW images is not a practical approach, as it may not effectively address the presence of calculus. Therefore, this study employed image-segmentation techniques to extract individual teeth from BW images, using image-segmentation algorithms and combines YOLO object-detection technology to locate individual teeth for the enhancement of symptoms.

**Figure 4:**
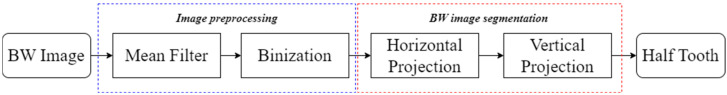
The flowchart used in single-tooth image segmentation.

This step aims to effectively separate the target of interest (individual tooth images) from the background (non-dental regions), as illustrated in Figure 4. This process involves multiple image-processing steps and identifies peaks and valleys for segmentation through accumulated pixel values. Considering that dental calculus symptoms often occur on both sides of the teeth, to enhance the effectiveness of CNN training, this study divided each tooth into two equal parts vertically.

A. Image Preprocessing

**Figure 5:**
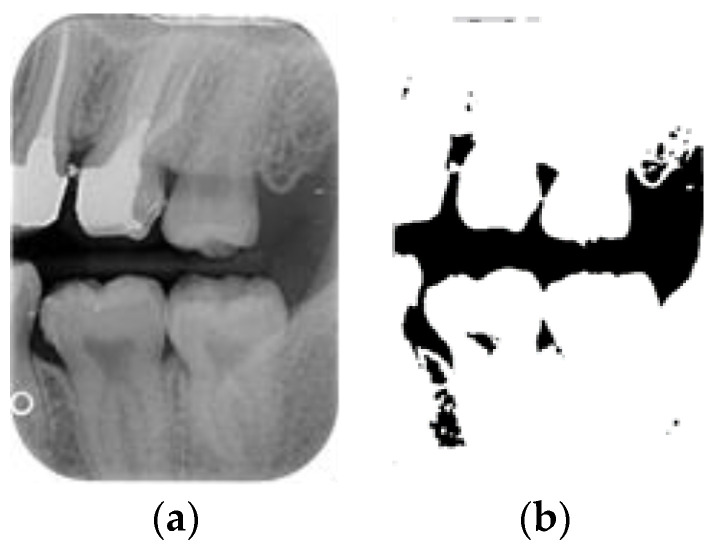
The BW image-preprocessing results: (**a**) mean filter; (**b**) binarization.

To obtain clearer images and to reduce noise, mean filtering was applied to the original images of the BW images in this study. Mean filtering is a form of linear filtering that computes the average pixel value within a window region and assigns this average value to the pixel at the center of the window. This method is simple and efficient, effectively blurring noise information. The formula for mean filtering is as shown in (1), and the filter results are shown in Figure 5a.

(1) $$Output\left(x,y\right)=\frac{1}{mn}\sum_{s=-a}^{a}\sum_{s=-b}^{b}Input(x+s,y+t)$$

After the mean filter-smoothing process, the BW images retained a significant amount of information. This study used image processing to simplify the image structure and reduce excessive information by converting the grayscale BW images into binary images containing only two-pixel values: 0 and 255. By setting a threshold of 170, pixels with values greater than 170 were set to 255, while pixels with values less than or equal to 170 were set to 0. The results are illustrated in Figure 5b. The binary images effectively reduced the complexity of the BW images, allowing for a clearer identification of key features and structures. This simplified representation facilitated further analysis and interpretation, enabling us to focus on specific areas of interest with greater precision and clarity. The streamlined visual data provided a solid foundation for subsequent quantitative and qualitative assessments, enhancing the overall efficacy of this study’s findings.

B. BW Image Pixel-Projection Algorithm

**Figure 6:**
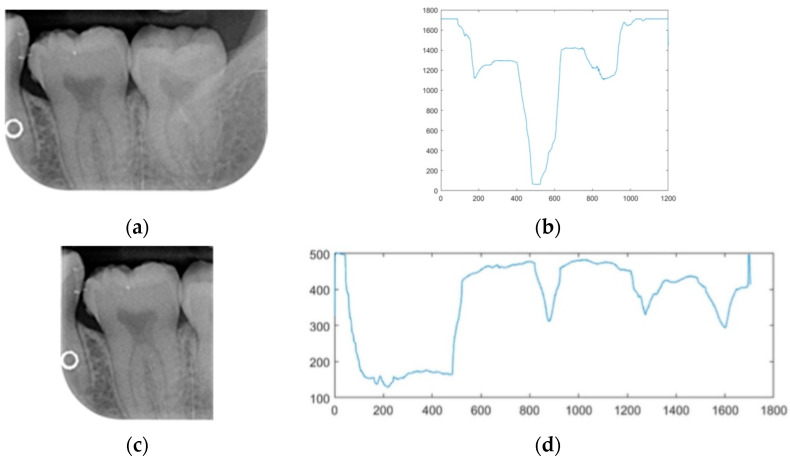
The results of pixel projection: (**a**) horizontal pixel projection; (**b**) horizontal pixel projection-coordinate graph; (**c**)vertical pixel projection; (**d**) vertical pixel projection-coordinate graph.

This study employed image segmentation to enhance the precision of diagnosing dental calculus symptoms by segmenting the BW image into individual teeth. At first, horizontal projection was utilized to detect the coordinates of the pixel accumulation with the lowest value in the BW image (refer to Figure 6b). Subsequently, based on these coordinates, the image was divided into upper and lower parts, as illustrated in the segmentation results shown in Figure 6a. Following the initial step, vertical projection was employed to identify the spaces between the teeth. A threshold of 400 for the total pixel values was established to facilitate the identification of multiple points falling below this threshold (refer to Figure 6d). Subsequently, when cropping the image, 50 pixels were subtracted from the smaller X coordinate, and 50 pixels are added to the larger X coordinate to prevent an inadvertent truncation of the tooth edges. The segmented individual teeth are depicted in Figure 6c.

### 2.3. Single-Tooth Image Enhancement

**Figure 7:**
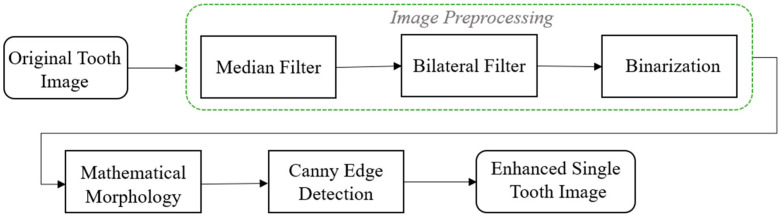
Image-enhancement flowchart.

Dental calculus typically appears on the surface of teeth or in the areas between teeth and gums, presenting as white or gray-white areas on X-ray images, with higher contrasts compared to surrounding teeth and tissues [29]. The visibility of dental calculus on BW images depends on factors such as its size, density, and location, as well as the angle and quality of the X-ray images. However, there may be challenges in discerning the signs of dental calculus. This study developed a dental calculus-enhancement algorithm tailored for BW images. The algorithm flowchart shown in Figure 7 integrates image-filter and morphological algorithms to enhance tooth edge structures in BW images, effectively highlighting dental calculus signs through edge-detection algorithms.

#### 2.3.1. Median Filter

BW image-segmentation results often contain a lot of noise, and dental calculus symptoms are not always clearly visible. Therefore, it is necessary to perform primary filtering on the noise in an image using a median filter. The median filter is an effective filtering method that removes noise while preserving image details and edge information, without causing excessive blurring of the image. This helps to improve the accuracy of dental calculus symptom detection. The process begins by selecting a pixel in the image and creating a window around it. The pixel values within the window are then sorted, and the median value is calculated. This median value is assigned to the pixel being processed. This process is repeated for each pixel in the image, resulting in a denoised image with preserved details and edges. The algorithm for the median filter used in this study is as shown in Algorithm 1:
**Algorithm 1.** Median Filter.**Input** $I_{i}$: filtering input image.        K: neighborhood kernel.        $K_{w},K_{h}$: kernel’s width and height. **Output** $I_{o}:\;$*filtering output image.*            $I_{o}\left[x,y\right]=median\{K\left[m,n\right]\times I_{i}\left[x-\left(n-\left[K_{w}/2\right]\right),y-(m-\left[K_{h}/2\right])\right]\}$ **Hint:** m $\in \left[0\ldots K_{h}-1\right],\;n\in \left[0\ldots K_{w}-1\right]$

#### 2.3.2. Bilateral Filter

This study introduced the bilateral filter technique to enhance the quality of individual tooth images. Compared to median filtering, a bilateral filter is more effective in handling images with different textures while preserving the clarity of image textures. This technique combines spatial- and pixel-value domain similarities, overcoming the limitations of traditional filtering methods in retaining texture details. The bilateral filter algorithm in Algorithm 2 computes the spatial distance between each pixel and its surrounding pixels, along with the difference in pixel values. It then uses these differences to calculate the weights of the pixels and applies these weights to each pixel in the image. It obtains the new pixel values by applying a weighted average of the pixel values in the surrounding region. A bilateral filter is useful for enhancing specific details in dental images, making the edges of the teeth clearer while preserving the natural texture of the image. Applying this technique can improve the accuracy of detecting dental calculus symptoms and can make tooth images more suitable for further analysis and processing.
**Algorithm 2.** Bilateral Filter.**Input**   $I_{i}$: filtering input image.          X: the coordinates of the current pixel.          Ω: the window center centered in X.          $F_{r}$: the range kernel for smoothing differences in intensities.          $G_{S}$: the spatial kernel for smoothing differences in coordinates.          W: normalization term between spatial closeness ($F_{r}$) and intensity difference ($G_{S}$). **Output**   $I_{o}\;:\;$*filtering output image.*                  $I_{o}\left(x\right)=\frac{1}{W}\sum_{X_{i}\in \Omega \;}I_{i}\left(x_{i}\right)F_{r}(\left\|{\;I}_{i}\left(x_{i}\right)-I_{i}\left(x_{i}\right)\right\|)G_{S}(\left\|x_{i}-x\right\|)$ **Hint:** $\;W=\sum_{X_{i}\in \Omega \;}F_{r}(\left\|{\;I}_{i}\left(x_{i}\right)-I_{i}\left(x_{i}\right)\right\|)G_{S}(\left\|x_{i}-x\right\|)$

#### 2.3.3. Binarization

**Figure 8:**
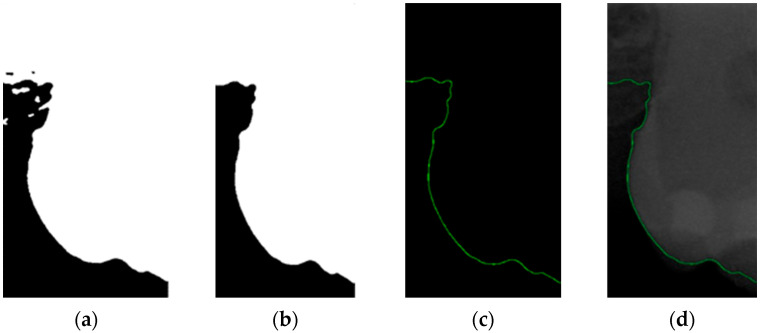
Image-enhancement results: (**a**) binarization; (**b**) mathematical morphology; (**c**) added green line represents canny; (**d**) overlap onto the original image.

The grayscale images of individual teeth obtained through two sets of image filters have pixel-intensity values ranging from 0 (black) to 255 (white). These images display intricate details and layers, allowing for a clear depiction of dental structural characteristics. Dental calculus located at the boundaries of teeth may not be distinctly recognizable in grayscale images. In this study, image binarization was used to simplify and enhance the visibility of teeth against the background to improve the detection of pathological features. Binarized images contain only two-pixel values, typically used to separate objects from the background, thereby enhancing the clarity of object contours and structures. Adaptive thresholding was applied in this study to ensure more accurate binarization results under varying illumination conditions. The binarization results, as shown in Figure 8a, demonstrate a clear contrast between the teeth and the background.

#### 2.3.4. Mathematical Morphology

This study utilized morphological operations to improve the success rate of edge detection. Morphological operations are fundamental operations in mathematical morphology, which are primarily used to enhance specific features of images such as edges, shapes, and structures that are widely applied in image processing. Opening and closing are the two main operations, and their formulas are as shown in (2) and (3). These operations are based on combinations of dilation and erosion. The opening operation removes small objects or spots from an image, while the closing operation fills in small holes or gaps in an image. The results of morphological operations are shown in Figure 8b.

(2) A○B=(A⊕B)⊖B

(3) A˙B=(A⊖B)⊕B

#### 2.3.5. Canny Edge Detection

Since filtered and binarized images alone cannot enhance the features of dental calculus, this approach does not effectively improve the accuracy of machine learning. Therefore, this study employed edge detection to identify the contours of tooth edges, enhancing the regions of interest and thereby improving the accuracy of machine learning. The canny edge-detection algorithm applies non-maximum suppression to each pixel, retaining pixels with local maximum gradient values, which can produce continuous and accurate edges while demonstrating good resistance to noise. The edge-detection results are represented in green, as shown in Figure 8c. Finally, these results are overlaid back onto the original image, as illustrated in Figure 8d.

### 2.4. CNN Training and Validation

CNN is a powerful deep learning model for visual data processing, and its importance in medicine is increasing [30]. It extracts key features from complex visual data and aids in model training through layers and adjustments. After refining the dental calculus image, CNN goes through training phases including database augmentation and tuning. The model is validated using untrained images of dental calculus to assess accuracy and reliability. Using CNN for dental calculus detection offers significant benefits, such as precise feature extraction and improved diagnostic accuracy. Experimental validation shows that the proposed CNN model achieves good results in determining the presence of dental calculus.

#### 2.4.1. Dataset Augmentation

**Figure 9:**
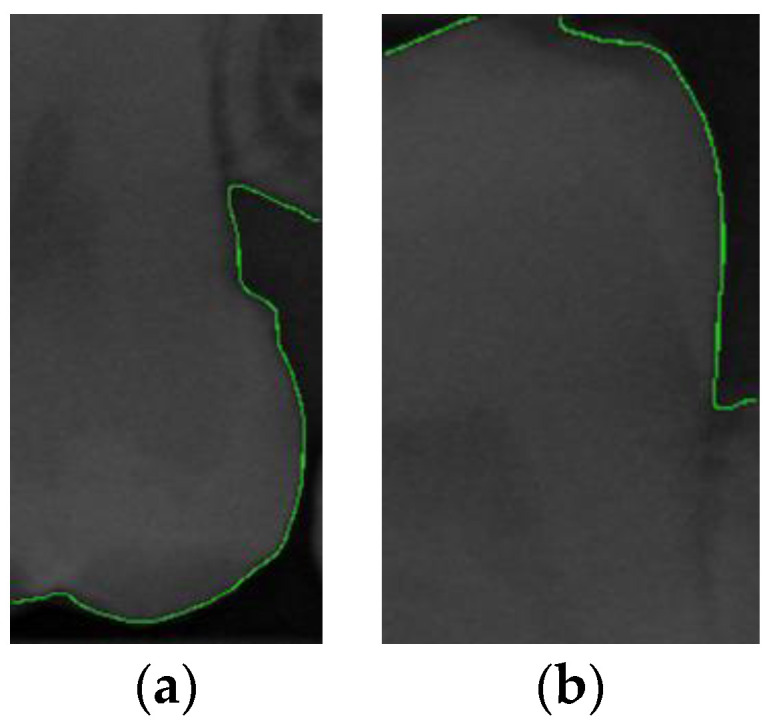
Data augmentation results. (**a**) Dental calculus. (**b**) Without dental calculus.

For CNN models, having an adequate amount of data is crucial to ensure effective learning and generalization performance. When the training sample size is limited, models are prone to overfitting or may fail to achieve good generalization performance. After applying the image-segmentation technique developed in this study, a total of 1340 images of individual teeth were obtained, including 428 images with dental calculus and 912 images without dental calculus. To balance the data quantity and to reduce overfitting issues, this study employed random image-flipping and mirroring techniques for data augmentation. This study set the dataset to include 670 images with dental calculus and 670 images without dental calculus. Dental calculus images were randomly selected for flipping or mirroring, while non-dental calculus images were also randomly selected to form the dataset for CNN training and validation. The detailed breakdown is presented in Table 4. A total of 80% of the image dataset was randomly chosen as the CNN training dataset. The remaining 20% of the dataset was used as the validation-image dataset. Moreover, the CNN training dataset was split into a 7:3 ratio for the training and testing datasets, and the training image as shown in Figure 9.

#### 2.4.2. Hyperparameter Tuning

During the training process of a CNN (Convolutional Neural Network), hyperparameters are manually set parameters that directly influence the model’s learning process and performance. Some common hyperparameters include the following:

1. Learning rate: This controls the updated speed during training. A higher learning rate can accelerate convergence but may lead to oscillations, while a lower learning rate may result in slow convergence.
2. Batch size: This represents the number of samples used to update the model parameters during each training iteration. A larger batch size can improve the training speed but increases memory requirements, whereas a smaller batch size may lead to unstable training.
3. Epoch: This represents the number of times the entire training dataset is traversed during training. Increasing the number of epochs allows the model to better learn from the data but may also lead to overfitting.

Adjusting hyperparameters requires finding suitable ranges, selecting an optimization method, and testing different combinations to choose the best set. This research identifies the optimal hyperparameter mix for dental calculus recognition in Table 5. Ensuring precise tooth identification and successful training, the dental panorama X-ray unit is shown in Table 6.

#### 2.4.3. CNN Model Training

CNN offers advantages in image processing by autonomously extracting abstract features, beneficial for detecting subtle features like dental calculus. CNN has fewer parameters, reducing computational costs and memory needs and addressing overfitting. This study uses four deep learning models within the CNN framework: GoogLeNet, ShuffleNet, Xception, and Inception_v3. These models were employed for processing image data. GoogLeNet, known for its Inception architecture, utilizes multi-scale convolution kernels to enhance performance. The CNN model training acceleration in this study was achieved using an Nvidia GeForce GTX 2070 GPU. Detailed hardware performance information is provided in Table 7. MATLAB and Deep Network Designer are key software tools for creating convolution network models.

## 3. Results

This research utilized a YOLO and CNN model to identify symptoms of dental calculus to reduce the workload of dentists and to minimize the chances of misdiagnosis. Image-enhancement methods were employed to improve the accuracy by enhancing characteristics that might not be clearly visible in the images.

A. YOLO Detects Single Teeth

YOLO is an artificial intelligence technology capable of detecting regions of interest (RoI) in images. This study used bounding boxes to locate features and extract positional information from them to extract these features. To evaluate the efficacy of AI model training, different standards are typically used for comparison. Commonly used standards in current research are shown below and were calculated based on the confusion matrix in Table 8, with the formulas illustrated in (4)–(7).

1. Precision: the proportion of all items detected as targets that are correctly classified as targets.
2. Recall: the proportion of all targets in the data that are correctly classified as targets, also called sensitivity.
3. mAP (mean average precision): the average of these average precision values across all classes, which is computed by plotting a precision–recall curve for each class and calculating the area under the curve (AUC).
4. Specificity: the proportion of targets that are actually not diseases that are tested as correct.

(4) $$Precision=\frac{T_{p}}{T_{p}+F_{p}}$$

(5) $$Recall(Sensitivity)=\frac{T_{p}}{T_{p}+F_{n}}$$

(6) $$mAP=\frac{\sum_{q=1}^{Q}\frac{1}{n}\sum_{rÎ\{0,0,1\ldots \ldots 1\}}P_{interp}(q)}{Q}$$

(7) $$Specificity=\frac{T_{N}}{F_{P}+T_{N}}$$

In the validation results, this study compared image-segmentation algorithms, and the results are shown in Table 9. It can be seen that the use of a YOLO object-detection model results in a better accuracy than that of a segmentation algorithm. Table 10 demonstrates that YOLOv8 achieves the highest precision and mAP values, reaching 97.48% and 99.37%, and Table 11 demonstrates a comparison with another research study. Meanwhile, YOLOv5 exhibits the best recall performance at 97.82%. This study also compares these results with those obtained using YOLO models in references [19,31]. The YOLO models proposed in this study show an improvement of up to 19% in feature detection and segmentation.

**Figure 10:**
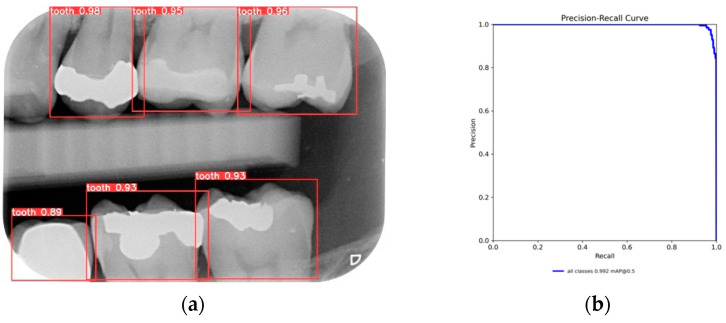
YOLO validation results: (**a**) YOLOv8 detect results; (**b**) YOLOv8 validation PR curve.

**Figure 11:**
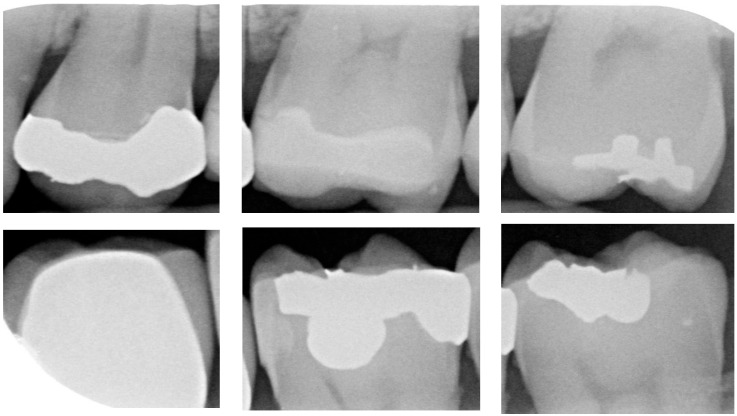
The results of tooth extraction based on YOLOv8.

After training the YOLO object-detection models, this study tested unlabeled, and untrained the BW images. The test results are illustrated in Figure 10. The four object models used in this study, along with the image-augmentation technique, successfully identified all the teeth of interest in the BW images. After the image-segmentation stage, the extracted regions were delineated by the red boxes in Figure 10a, and the PR curve is shown in Figure 10b. The results extracted are depicted in Figure 11, effectively capturing the individual teeth and their edges and enabling further image-enhancement and CNN training steps.

B. Dental Calculus Classification Result

**Figure 12:**
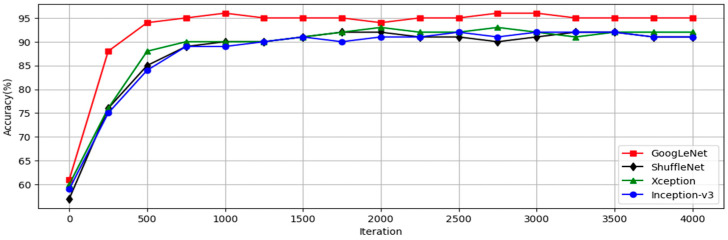
CNN training process.

**Figure 13:**
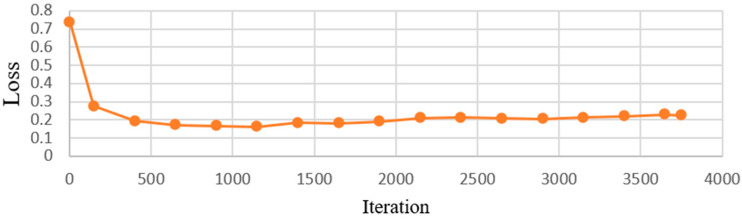
GoogLeNet loss process.

This research utilized a CNN model to identify symptoms of dental calculus to reduce the workload of dentists and to minimize the chances of misdiagnosis. Image-enhancement methods were employed to improve the accuracy by enhancing characteristics that might not be clearly visible in the images. With the CNN model-training accuracy shown in Figure 12, it can be seen that the accuracy of each model increases rapidly within the first few hundred iterations and then gradually stabilizes. The GoogLeNet model shows the most outstanding performance, achieving the highest accuracy at all iteration counts, while the other models stabilize at similar accuracy levels after approximately 1000 iterations. This indicates that GoogLeNet performs better in this binary classification task. Figure 13 shows the GoogLeNet loss function curves during training. Table 12 provides training process details of GoogLeNet. The results offer insight into the model’s training progress and effectiveness, demonstrating successful dental calculus symptom identification. Image-enhancement techniques improved the performance, as visualized through the training convergence and loss function curves, highlighting the model’s effectiveness in diagnostics and patient care.

This study trained four popular CNN models, and their training outcomes are presented in Table 13. It is evident that when trained on single-tooth diseases without image enhancement, the highest accuracy reached was 75%. Dental calculus characters are difficult to see in the images. This study used image-enhancement methods to improve training results, which resulted in significant improvements. For instance, using GoogLeNet as an example, its accuracy reached 96.11%, marking an approximately 20% increase compared to the original results. It is worth noting that even the ShuffleNet model, despite having lower accuracy, achieved a dental calculus detection accuracy of 91.58%. 

This study conducted validations using 8 untrained images to validate the integrity of the training results, as shown in Table 14, and to provide the comprehensive evaluation with 200 BW images with YOLOv8 in Table 15. The validation process involved steps such as YOLO image segmentation, image enhancement, and CNN symptom classification. By comparing the results with the Ground truth obtained through discussions with multiple dentists, it was found that all eight teeth could accurately determine the presence of dental calculus, with an accuracy exceeding 90% for each. The performance improvement of GoogLeNet, compared to the latest research by [15] in symptom-detection accuracy, is also approximately 14%, which is shown in Table 16.

## 4. Discussion

This study addresses the critical goal of reducing the burden on dentists and minimizing the risk of misdiagnosis in dental calculus detection. By leveraging the strengths of BW images, which excel in capturing tooth tissue and lesion characteristics [32], this study aimed to improve the identification of dental calculus, a subtle yet significant symptom that often requires careful examination. The implications of this research are far-reaching, particularly in busy clinical settings where time constraints and fatigue may contribute to an oversight of subtle dental plaque, potentially impacting patient treatment plans and oral health outcomes. In comparison to prior research utilizing traditional radiographic imaging, this study demonstrated a substantial enhancement in mAP by 19% through the adoption of a different object-detection model and preprocessing image-enhancement techniques [33]. Moreover, when compared to the direct utilization of YOLO_v5 for dental calculus detection in BW images [34], there was a minimum 15% increase in mAP. Using the YOLOv8 model and image preprocessing as the tool for detecting single teeth achieves an accuracy of 97%, highlighting the efficacy of the proposed methodology. The integration of AI models resulted in a notable accuracy improvement of 82%, effectively reducing errors in dental calculus detection compared to previous approaches [15]. Building upon these achievements, this study implemented the segmentation of dental BW images to identify single-tooth images and applied enhancements to boost diagnostic accuracy. The utilization of GoogLeNet yielded a final accuracy of 96.11%, reflecting a noteworthy 10% enhancement over previous methods [31]. The comprehensive comparison in dental calculus is shown in Table 17. However, it is essential to acknowledge the limitations of the current study. While it successfully identifies dental calculus features in BW images, issues related to potential tartar and other dental problems remain unresolved. Looking ahead, future research endeavors should focus on exploring alternative image-processing techniques, particularly in the interdental area, to effectively extract and address these additional challenges. By broadening the scope of investigation and considering diverse methodologies, researchers can continue to advance the field of dental diagnostics, contributing to improved patient care and oral health outcomes. This study did not address the detection of dental caries or other lesions, which can also impact overall oral health. Furthermore, the dataset used for training and validation may not fully represent the diversity of dental cases encountered in different populations. This limitation could affect the generalizability of this research. Lastly, this study focused on single-tooth segmentation, which, although effective, may not fully capture the complexity of dental structures in multi-tooth contexts. The comprehensive comparison in dental calculus is shown in Table 17.

## 5. Conclusions

This research represents a significant breakthrough in the diagnosis of dental calculus symptoms. In the future, clinical practitioners can enhance their diagnostic and treatment capabilities in dental calculus by utilizing the assistance provided by this study, thereby improving the quality of healthcare. It can achieve an accuracy of more than 92% in classifying disease symptoms, and it can also achieve a 10% optimization compared with current research. In future research, we plan to analyze a large database of images and utilize Generative Adversarial Networks (GANs) for image enhancement, replacing traditional image preprocessing steps. Through GANs, the model can automatically learn and generate more medically meaningful image features, thereby reducing manual intervention in the model and improving the efficiency of image enhancements. This will help shorten the model’s decision-making time while providing more comprehensive medical diagnostic support, contributing significantly to dental healthcare services. This study proposes that future research should focus on investigating the application of advanced deep learning models to enhance diagnostic accuracy and comprehensiveness. This could lead to improvements in the detection and analysis of various dental conditions. Moreover, exploring the use of generative AI could provide significant advancements in this field. Additionally, enhancing image quality by reducing noise could lead to more detailed dental image information. These advancements may enable more precise identification and the development of more effective treatment plans.

## Figures and Tables

**Table 1:** YOLO image dataset distribution.

Category	Image Number
Training dataset	130
Testing dataset	70
Validation dataset	235

**Table 2:** YOLO architecture comparison.

Model	Backbone	Neck	Head
YOLOv4	CSPDarknet53	PANet	YOLO head
YOLOv5	Custom CSPNet	PANet	YOLO head
YOLOv7	Extended CSPNet	E-ELAN	YOLO head with decoupled head
YOLOv8	Mixture of experts	Advanced PANet	YOLO head with decoupled head

**Table 3:** YOLO model training hyperparameters.

	Epochs	Batch Size	Learning Rate
YOLOv4	30	1	0.001
YOLOv5	100	8	0.01
YOLOv7	228	4	0.01
YOLOv8	50	8	0.01

**Table 4:** Distribution of CNN-model-image dataset.

The number of datasets before and after dataset augmentation											
				Before				After			
Dental calculus				428				670			
Without dental calculus				912				670			
The number of datasets in the CNN model											
			Training			Testing			Validation		
Image number			750			322			268		

**Table 5:** The hyperparameter used in the CNN model training.

Hyperparameter	Value
Learning Rate	0.0001
Batch Size	4
Epochs	30
Validation Frequency	50

**Table 6:** Dental panorama X-ray unit.

Exposure Time	Incrementally adjustable from ≤ 0.03 to 3.2 s
X-Ray generator	High-frequency generator for a constant high
X-Ray tube focal spot	≤ 0.5 mm
Image developing speed	≤ 5 s
Sensor size	31.3 mm × 44.5 mm
Image format	DCI

**Table 7:** The hardware and software platform.

Hardware	Specifications	Manufacturer	Software	Version
CPU	Intel(R) core i7-8700	Intel, California, United States	MATLAB	R2023b
GPU	NVIDIA GeForce GTX 2070	NVIDIA, California, United States	Deep Network designer	14.5
DRAM	32 GB	ADATA, New Taipei City, Taiwan	PyTorch	1.8

**Table 8:** Confusion matrix.

		Ground Truth Value	
		True	False
Predicted Value	True	Tp (True positive)	Fp (False positive)
	False	Fn (False negative)	Tn (True negative)

**Table 9:** Comparison of image-segmentation methods between algorithms and YOLO.

Method	Algorithm	YOLOv4	YOLOv5	YOLOv7	YOLOv8
Accuracy	82.4%	91.8%	94.72%	95.66%	96.99%

**Table 10:** YOLO training results.

		Precision	Recall (Sensitivity)	Specificity	mAP
This Study	YOLOv4	92.4%	92.4%	91.77%	92.00%
	YOLOv5	95.86%	97.82%	96.85%	98.96%
	YOLOv7	97.2%	97.65%	98.10%	99.24%
	YOLOv8	97.48%	96.81%	98.25%	99.27%
Compared with [19]		96.91%	82.32%	X	82.77%
Compared with [31]		82.36%	78.38%	X	80.37%

**Table 11:** YOLO comparison with another research study.

	This Study	Method in [31]		
Model	YOLOv8	YOLOv8S	YOLOv8M	YOLOv8L
Function	Tooth Detction			
Precision	0.975	0.956	0.914	0.924
Recall	0.968	0.945	0.942	0.977
mAP50	0.993	0.921	0.909	0.935

**Table 12:** GoogLeNet training process in every 5 epochs.

Epoch	Iteration	Time Elapsed	Mini Batch	Testing
1	100	00:00:32	62.50%	86.23%
5	600	00:02:48	100.00%	92.81%
10	1250	00:05:49	100.00%	94.01%
15	1850	00:08:37	100.00%	94.91%
20	2500	00:11:48	100.00%	94.61%
25	3100	00:14:53	100.00%	94.91%
30	3750	00:18:36	100.00%	96.11%

**Table 13:** CNN model-training accuracy.

	Original	Image Enhancement
GoogLeNet	75.00%	96.11%
ShuffleNet	72.12%	91.58%
Xception	68.33%	92.38%
Inception-v3	62.92%	91.62%

**Table 14:** The dental calculus image-validation results.

Ground truth: Dental calculus	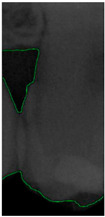	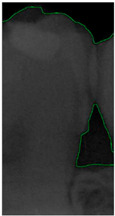	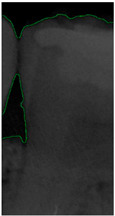	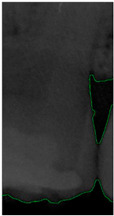
Accuracy	97.13%	99.99%	93.92%	92.73%
Ground truth: others	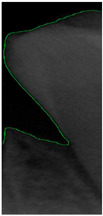	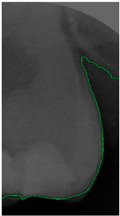	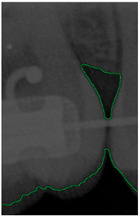	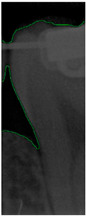
Accuracy	96.17%	99.83%	97.25%	91.60%

**Table 15:** Validation results with a dataset of 200 images.

Model	Epochs	Batch Size	Learning Rate	Precision	Recall	mAP50
YOLOv8	50	8	0.01	0.977	0.965	0.992

**Table 16:** CNN training result comparison.

GoogLeNet	This Study		Method in [15]
	Original	Image Enhancement	
	75.00%	96.11%	82.2%

**Table 17:** Comprehensive comparison in dental calculus.

Function	Image Detection		
Method	This study	[33]	[34]
mAP	99.27%	80.37%	79.72%
Function	Image classification		
Method	This study	[15]	[31]
Accuracy	96.11%	82.2%	85.24%

## Data Availability

Based on the IRB and the agreement with Chang Gung Memorial Hospital, its database and related data cannot be disclosed.
